# Racial-Ethnic Composition of Primary Care Practices and Comprehensive Primary Care Plus Initiative Participation

**DOI:** 10.1007/s11606-023-08160-0

**Published:** 2023-03-20

**Authors:** Karl Rubio, Taressa K. Fraze, Salma Bibi, Hector P. Rodriguez

**Affiliations:** 1 Division of Health Policy and Management, School of Public Health, University of California, Berkeley, Berkeley, CA USA; 2 Department of Family and Community Medicine, University of California, San Francisco, San Francisco, CA USA

**Keywords:** delivery system reform, chronic care management, independent physicians, health systems, primary care practices

## Abstract

**Background:**

It remains unclear whether the racial-ethnic composition or the socioeconomic profiles of eligible primary care practices better explain practice participation in the Centers for Medicare and Medicaid Services’ (CMS) Comprehensive Primary Care Plus (CPC+) program.

**Objective:**

To examine whether practices serving high proportions of Black or Latino Medicare fee-for-service (FFS) beneficiaries were less likely to participate in CPC+ in 2021 compared to practices serving lower proportions of these populations.

**Design:**

2019 IQVIA OneKey data on practice characteristics was linked with 2018 CMS claims data and 2021 CMS CPC+ participation data. Medicare FFS beneficiaries were attributed to practices using CMS’s primary care attribution method.

**Participants:**

11,718 primary care practices and 7,264,812 attributed Medicare FFS beneficiaries across 18 eligible regions.

**Methods:**

Multivariable logistic regression models examined whether eligible practices with relatively high shares of Black or Latino Medicare FFS beneficiaries were less likely to participate in CPC+ in 2021, controlling for the clinical and socioeconomic profiles of practices.

**Main Measures:**

Proportion of Medicare FFS beneficiaries attributed to each practice that are (1) Latino and (2) Black.

**Key Results:**

Of the eligible practices, 26.9% were CPC+ participants. In adjusted analyses, practices with relatively high shares of Black (adjusted odds ratio, aOR = 0.62, *p* < 0.05) and Latino (aOR = 0.32, *p* < 0.01) beneficiaries were less likely to participate in CPC+ compared to practices with lower shares of these beneficiary groups. State differences in CPC+ participation rates partially explained participation disparities for practices with relatively high shares of Black beneficiaries, but did not explain participation disparities for practices with relatively high shares of Latino beneficiaries.

**Conclusions:**

The racial-ethnic composition of eligible primary care practices is more strongly associated with CPC+ participation than census tract–level poverty. Practice eligibility requirements for CMS-sponsored initiatives should be reconsidered so that Black and Latino beneficiaries are not left out of the benefits of practice transformation.

**Supplementary Information:**

The online version contains supplementary material available at 10.1007/s11606-023-08160-0.

## INTRODUCTION

The Centers for Medicare and Medicaid Services’ (CMS) Comprehensive Primary Care Plus (CPC+) program was the nation’s largest federally sponsored primary care transformation initiative.^1^ CPC+ is an “advanced” primary care program that has the potential to improve quality of care and reduce total spending for adult patients with chronic conditions, particularly Medicare beneficiaries. The Comprehensive Primary Care initiative, a predecessor of CPC+ , led to improvements in primary care delivery and reductions in emergency department visits.^2^ CPC+ provided independent and safety net practices with the opportunity to participate in advanced payment models without having to take on significant financial risk.^3^

Descriptive analyses of early (2017) area-level CPC+ participation patterns indicate that compared with areas with CPC+ practices, areas without CPC+ practices were characterized by disadvantages, including a lower median income ($54,303 vs $59,573) and a higher share of households living in poverty (23% vs 17%).^4^ Analyses of 2019 participation data indicate that primary care practices participating in CPC+ served relatively low shares of Black Medicare fee-for-service (FFS) beneficiaries compared to non-participating eligible physician practices in the 18 CPC+ geographic regions.^5^

Evidence indicates that racial and ethnic minority Medicare beneficiaries receive lower quality of care than White beneficiaries and have less access to primary and specialty care.^6^ Early results from CPC+ indicate that Medicare beneficiaries attributed to CPC+ practices reported better experiences of primary care than Medicare beneficiaries attributed to non-participating practices. There were no racial or ethnic differences in CPC+ effects, suggesting that Medicare beneficiaries of diverse backgrounds may benefit equally from practice participation in the initiative.^7^

CPC+ practices were encouraged by CMS to invest in comprehensive care capabilities such as medication management and extended hours,^8^ but the program was not initially designed with advancing equity as a priority when recruiting practices or designing program incentives.^9^ The CPC+ program had extensive eligibility requirements. Concierge practices, rural health clinics, federally qualified health center (FQHC), or a participant in any Medicare accountable care organization (ACO) other than the CMS Medicare Shared Savings Program or Transforming Clinical Practices Initiative (TCPI),^10^ a CMS collaborative and peer-based learning program, was not eligible to participate in CPC+. In addition, practices needed to demonstrate that (1) primary care was 40% or more of Medicare FFS activity, (2) practice revenue from Medicare and other participating CPC+ payers was at least 45% of the total revenue and had sufficient volume defined as 125 or more attributed Medicare FFS beneficiaries, (3) certified health information technology and electronic health record technology were used, and (4) the practice assigned patients to a provider panel, provided 24/7 access for patients, had non-physician team members deliver some clinical care, and supported quality improvement. These extensive requirements may have the unintended effect of creating participation barriers for practices serving high shares of Black and Latino beneficiaries across the CPC+ regions.

The Biden administration outlined an ambitious plan to advance equity, and CMS also plans to improve the reach of federally sponsored practice transformation initiatives by improving the diversity of practices and CMS beneficiaries who benefit from practice transformation. CMS also plans to improve the collection and analysis of equity data, monitor and evaluate impacts on equity, and operationalize equity performance into new payment and care delivery models.^11^ To inform these efforts, we examine whether primary care practices with relatively high shares of Black and Latino Medicare FFS beneficiaries were less likely to participate in CPC+ compared to primary care practices with lower shares of these groups, controlling for regional differences in participation rates, clinical profiles of practices, and census tract–level poverty. We advance evidence by analyzing 2021 CPC+ participation data, examining Black and Latino beneficiary as separate main independent variables, and using multivariable regression analyses to assess the relative association of practice racial-ethnic composition and census tract–level poverty with CPC+ participation.

## METHODS

### Data Sources

To identify CPC+ practices, we analyzed CPC+ participant data from CMS which included 3428 practices. We used the participant list produced by CMS on April 10, 2021, which was the most recent data available from CMS at the time of analyses. Given past documented differences between CPC+ tracks (track 1 vs. track 2)^5^ and sample size considerations, we focused on overall practice participation differences rather than track-specific differences. To characterize the practices, we linked the CMS participant file to the 2019 IQVIA OneKey file, a commercially available database of practice structural characteristics. The OneKey file used data from the American Medical Association, public sources, and proprietary data collection methods to describe physician practices, including information about practices such as practice ownership, size, and addresses. The OneKey file also associated National Provider Identifiers for practicing clinicians with practices in the 18 CPC+ regions, which include Arkansas, Colorado, Hawaii, Greater Kansas City Region of Kansas and Missouri, Louisiana, Michigan, Montana, Nebraska, New Jersey, Greater Buffalo Region of New York, North Hudson-Capital Region of New York, North Dakota, Ohio and Northern Kentucky Region, Oklahoma, Oregon, Greater Philadelphia Region of Pennsylvania, Rhode Island, and Tennessee. The methods description in the Supplemental Material and eFigure 1 detail the sample restriction to eligible regions using IQVIA OneKey data and the CMS CPC+ participant file. We identified CPC+ practices using fuzzy string matching,^12^ while restricting the parameters to match within eligible states (eFigure 2). Then, we constructed a comparison group of eligible practices in the CPC+ regions that were not CPC+ participants (eFigure 3). Our final analytical sample included 11,718 practices, 3152 (27%) of which were CPC+ participants and 8566 (73%) were not CPC+ participants.

### Measures

#### Outcome Measure

Practice participation in CPC+ is a binary indicator of practice participation in the CPC+ initiative during 2021.

#### Main Independent Variables

##### Proportion of Latino Medicare FFS Beneficiaries

For each practice, we calculated the proportion of Latino beneficiaries as the count of Medicare FFS beneficiaries attributed to each practice who are Latino divided by the total count of Medicare FFS beneficiaries attributed to the practice.

##### Proportion of Black Medicare FFS Beneficiaries

For each practice, we calculated the proportion of Black beneficiaries as the count of Medicare FFS beneficiaries who are Black divided by the total count of Medicare FFS beneficiaries attributed to the practice.

#### Control Variables

Control variables include census tract–level poverty defined as the proportion of beneficiaries that resided in a census tract with 20% or more of residents with incomes at or below 100% of the federal poverty level, and the median household income of attributed beneficiaries’ ZIP codes. Beneficiary profiles of practices were also included as control variables which are practice-level mean patient age, proportion of female beneficiaries, hierarchical condition category (HCC) risk-adjustment factor score, and dual eligibility for Medicare and Medicaid to account for differences in patient morbidity and individual dummy variables of HCCs for the diagnosis of frailty. HCC codes are used by CMS as part of risk-adjustment models that assign each patient a score to reflect their projected risk for high healthcare utilization.^13^ Frailty was defined as beneficiaries having 2 or more frailty indicators (abnormality of gait, malnutrition, failure to thrive, cachexia, debility, difficulty in walking, fall, muscular wasting and disuse atrophy, muscle weakness, decubitus ulcer of skin, senility without mention of psychosis, or durable medical equipment use [cane, walker, bath equipment, and commode]).^14^

Practice characteristics included as control variables are practice ownership which is categorized as (a) independent, (b) medical group owned, and (c) hospital or health system owned. Control variables also included accountable care organization participation and standardized values of the total numbers of primary care physicians, specialists, and advanced-practice clinicians (physician’s assistants, nurse practitioners, and clinical nurse specialists).

### Analyses

First, we compared descriptive characteristics of practices participating in CPC+ and comparison practices using IQVIA OneKey data. Second, we analyzed the Medicare FFS data, collapsed at the practice level, to compare beneficiary characteristics between CPC+ practices and comparison practices. For both analyses, *t* tests were used to examine whether differences between CPC+ and comparison group practices were statistically significant in unadjusted analyses. Third, we conducted a series of three multivariable logistic regression models to examine the extent to which the proportion of Latino or Black beneficiaries is associated with CPC+ participation. Model 1 includes only the main independent variables, and model 2 includes the full set of control variables in addition to the main independent variables. Model 3, our main model, includes all model 2 covariates plus state fixed effects to account for the clustering of practices within states and to control for unmeasured state effects that could impact CPC+ participation. We also estimated predicted probabilities of CPC+ participation for practices with varying practice concentrations of Black and Latino beneficiaries using estimates from the main model (model 3) to illustrate the extent of exclusion of Latino and Black Medicare FFS beneficiaries from the benefits of CPC+. As a sensitivity analysis (model 4), we re-estimated model 3 restricted to CPC+ (*n* = 2742) and non-CPC+ (*n* = 5435) practices with 125 or more total beneficiaries to examine the impact of this program participation requirement. The restriction disproportionately removed non-CPC+ practices; 13% of CPC+ practices were restricted out, while 36% of non-CPC+ practices were restricted out. We considered *p* < 0.05 as the threshold for statistical significance for all analyses.

## RESULTS

### Descriptive Analyses

The participation rate for eligible practices across the 18 CPC+ regions was 26.9%. In unadjusted analyses (Table 1), all practice characteristics assessed differed for CPC+ and non-CPC+ practices; CPC+ practices have more physicians (primary care and specialist physicians) (6.7 vs. 3.9, *p* < 0.001), advanced-practice clinicians (2.5 vs. 1.8, *p* < 0.001), and specialist physicians (2.2 vs. 1.4, *p* < 0.001) compared to non-CPC+ practices. CPC+ practices were much less likely to be independently owned (27.6% vs. 55.3%, *p* < 0.001) than non-CPC+ practices. Non-CPC+ practices had higher proportions of Black (12.1% vs. 7.1%, *p* < 0.001), Latino (3.9% vs. 3.1%, *p* < 0.001), and high-poverty (23.1% vs. 16.6%) beneficiaries than CPC+ practices.

**Table 1:** Patient and Practice Characteristics, by Comprehensive Primary Care Plus Participation Status

	Overall	Non-CPC+ practice	CPC+ participant practice	Effect size and *p* value for difference
Practice *N*	11,718	8566	3152	
Patient profiles of practices				
Age				
Under 65 years	18.4%	19.9%	14.2%	0.057^†^
65 to 69 years	26.9%	26.8%	27.4%	−0.007^†^
70 to 74 years	20.9%	20.4%	22.2%	−0.018^†^
75 to 79 years	14.3%	14.0%	15.2%	−0.012^†^
80 to 84 years	9.5%	9.3%	10.1%	−0.008^†^
Over 85 years	10.0%	9.6%	10.9%	−0.012^†^
Female	57.2%	56.9%	58.0%	−0.011^†^
Race/ethnicity				
White	79.2%	77.6%	83.6%	−0.060^†^
Black	10.8%	12.1%	7.1%	0.050^†^
Latino	3.7%	3.9%	3.1%	0.008^†^
Black and Latino	14.5%	16.0%	10.2%	0.059^†^
Asian/Pacific Islander, American Indian/Alaska Native, and others	6.3%	6.3%	6.2%	0.001
Dually eligible for Medicare and Medicaid	6.6%	7.4%	4.4%	0.030^†^
Disabled	27.1%	29.1%	21.9%	0.072^†^
Hierarchical condition categories risk-adjustment factor (mean, SD)	1.09 [0.43]	1.10 [0.45]	1.06 [0.37]	0.039^†^
Congestive heart failure	9.4%	9.6%	9.0%	0.006^†^
Coronary artery disease	4.8%	4.9%	4.6%	0.003^†^
Diabetes	27.8%	28.6%	25.7%	0.029^†^
Cancer	8.7%	8.4%	9.5%	−0.011^†^
Chronic obstructive pulmonary disease	11.0%	11.4%	9.8%	0.016^†^
End-renal stage disease	1.2%	1.3%	1.0%	0.003^†^
Frail elder	4.3%	4.4%	4.1%	0.003^*^
Any mental illness	25.2%	25.6%	24.0%	0.017^†^
Died in 2018	3.3%	3.4%	3.0%	0.004^†^
Census tract–level poverty	21.4%	23.1%	16.6%	0.065^†^
ZIP code–level annual median household income (mean, SD)	$55,721 [$17,054]	$54,303 [$17,122]	$59,573 [$16,259]	− 52,670^†^
Total spending (mean, SD)	$10,049 [$7123]	$10,248 [$7218]	$9510 [$6831]	737^†^
Acute care/clinical access payments (mean, SD)	$3208 [$3484]	$3268 [$3095]	$3044 [$4364]	223^†^
Procedure payments (mean, SD)	$1493 [$926]	$1495 [$1020]	$1488 [$602]	7.564
Evaluation and management payments (mean, SD)	$1265 [$837]	$1279 [$891]	$1,28 [$667]	51^†^
Other payments (mean, SD)	$276 [$1042]	$307 [$1185]	$191 [$460]	116^†^
Practice characteristics				
Physicians (mean, SD)	4.65 [13.0]	3.90 [12.9]	6.69 [12.9]	−2.791^†^
Advanced-practice clinicians (mean, SD)	2.00 [3.5]	1.81[3.3]	2.51 [4.0]	−0.694^†^
Primary care physicians (mean, SD)	3.01 [5.7]	2.47 [5.2]	4.47 [6.6]	−2.009^†^
Specialists (mean, SD)	1.64 [9.3]	1.43 [9.4]	2.21 [9.0]	−0.782^†^
Practice size				
One physician	33.1%	38.1%	19.5%	0.186^†^
2–5 physicians	43.4%	40.9%	50.4%	−0.095^†^
6–10 physicians	9.4%	7.4%	14.7%	−0.073^†^
>10 physicians	7.9%	5.8%	13.5%	−0.077^†^
Practice ownership				
System or hospital	40.7%	35.2%	55.6%	−0.205^†^
Medical group	11.5%	9.5%	16.7%	− 0.072^†^
Independent	47.8%	55.3%	27.6%	0.277^†^
Rural–urban commuting area (RUCA)				
Isolated rural	4.6%	5.5%	2.0%	0.035^†^
Small town	6.8%	7.9%	3.9%	0.040^†^
Micropolitan	11.9%	12.8%	9.5%	0.033^†^
Metropolitan	76.7%	73.8%	84.5%	− 0.107^†^
Part of an accountable care organization (ACO)	21.9%	18.5%	31.0%	− 0.125^†^

Notes: The values displayed for *t* tests are the differences in the means across the groups

^*^p < 0.01, ^†^*p* < 0.001

### Adjusted Analyses

In regression analyses that only include our main independent variables, practices with relatively higher shares of Black beneficiaries (adjusted odds ratio, aOR = 0.177, *p* < 0.001) and Latino beneficiaries (aOR = 0.326, *p* < 0.001) had much lower odds of CPC+ participation (Table 2, Model 1). When controlling for beneficiary and practice characteristics, the association of practice concentration of Black (aOR = 0.482, *p* < 0.001) and Latino (aOR = 0.619, *p* < 0.106) beneficiaries is slightly attenuated (model 2). When state fixed effects were included for our main model (model 3), the odds ratio for the practice proportion of Black beneficiaries continued to attenuate (aOR = 0.622, *p* < 0.1), or moved closer to 1.0, while the odds ratio for the practice proportion of Latino beneficiaries became smaller (aOR = 0.317, *p* < 0.01), or moved further away from 1.0. eFigure 4 summarizes the high variation in participation rates among primary care practices within eligible regions of each CPC+ state.

**Table 2:** Multivariable Analyses: Practice Concentration of Black and Latino Medicare Fee-for-Service Beneficiaries and Comprehensive Primary Care Plus Program Participation

	Adjusted odds ratio			
	Model 1	Model 2	Model 3 (main model)	Model 4
Race/ethnicity				
White (reference)	–	–	–	–
Black	0.177^‡^ (0.106, 0.295)	0.482^‡^ (0.342, 0.679)	0.622^*^ (0.429, 0.893)	0.73 (0.453, 1.145)
Latino	0.326^‡^ (0.191, 0.556)	0.619 (0.342, 1.109)	0.317^†^ (0.16, 0.631)	0.547 (0.225, 1.325)
Asian/Pacific Islander, American Indian/Alaska Native, and others		0.9 (0.656, 1.254)	0.574^*^ (0.374, 0.9)	0.732 (0.435, 1.297)
Age				
Under 65 years (reference)		–	–	–
65 to 69 years		1.625 (0.934, 2.816)	3.688^‡^ (2.498, 5.387)	7.187^‡^ (0.435, 1.297)
70 to 74 years		4.053^‡^ (0.149, 10.958)	9.824^‡^ (4.964, 19.061)	233.5^‡^ (45.28, 1113.08)
75 to 79 years		3.317^‡^ (1.667, 6.551)	8.122^‡^ (4.334, 14.849)	1.613 (0.234, 10.512)
80 to 84 years		2.634^*^ (1.209, 5.651)	6.465^‡^ (3.673, 10.953)	4.124 (0.359, 43.334)
Over 85 years		5.479^‡^ (2.744, 10.812)	9.123^‡^ (4.679, 17.219)	5.004^*^ (1.038, 5.425)
Female		1.806^†^ (1.243, 2.621)	1.97^‡^ (1.329, 2.905)	2.661^‡^ (1.303, 5.425)
Dually eligible for Medicare and Medicaid		0.011^‡^ (0.003,0.043)	0.038^‡^ (0.014, 0.099)	0.017^‡^ (0.002, 0.116)
HCC score, standardized		1.003 (0.94, 1.07)	0.99 (0.929, 1.055)	1.089 (0.961, 1.234)
Frail elder		0.184^‡^ (0.066, 0.514)	0.266^*^ (0.095, 0.756)	0.176^*^ (0.035, 0.879)
Census tract–level poverty		0.922 (0.71, 1.198)	0.881 (0.665, 1.17)	0.982 (0.722, 1.356)
ZIP code–level annual median household income, standardized		1.14^‡^ (1.078, 1.203)	1.006 (0.944, 1.07)	0.995 (0.919, 1.073)
Number of physicians, standardized		2.248^‡^ (1.769, 2.856)	2.281^‡^ (1.787, 2.911)	2.251^‡^ (1.769, 2.855)
Specialists, standardized		0.443^‡^ (0.348, 0.564)	0.428^‡^ (0.333, 0.55)	0.431^‡^ (0.337, 0.554)
Advanced-practice clinicians, standardized		1.199^‡^ (1.136, 1.265)	1.258^‡^ (1.175, 1.346)	1.23^‡^ (1.157, 1.308)
Practice ownership				
Independent (reference)		–	–	–
Health system or hospital		2.758^‡^ (2.025, 3.664)	2.776^‡^ (2.012, 3.62)	2.653^‡^ (1.91, 3.29)
Medical group		3.367^‡^ (2.338, 4.769)	3.389^‡^ (2.331, 4.743)	3.275^‡^ (2.241, 4.413)
Part of an accountable care organization (ACO)		0.974 (0.945, 1.06)	0.944 (0.912, 1.03)	0.892 (0.856, 1.023)
Observations	11,718	11,718	11,718	8195
Pseudo *R*^2^	0.0148	0.111	0.166	0.16

Model 1 includes only the main independent variables, model 2 includes the main independent variables and covariates, and model 3 includes the main independent variables, covariates, and state fixed effects. Model 3 is our main model. Model 4 is a sensitivity analysis related to our final model (model 3), but restricted to practices with greater than 125 attributed beneficiaries (*n* = 8195), which includes 2742 CPC+ and 5453 non-CPC+ practices, which represents 87% and 64% of the study’s original analytic sample, respectively. 95% confidence intervals in parentheses

^*^p < 0.05, ^†^*p* < 0.01, ^‡^*p* < 0.001

**Figure 1:**
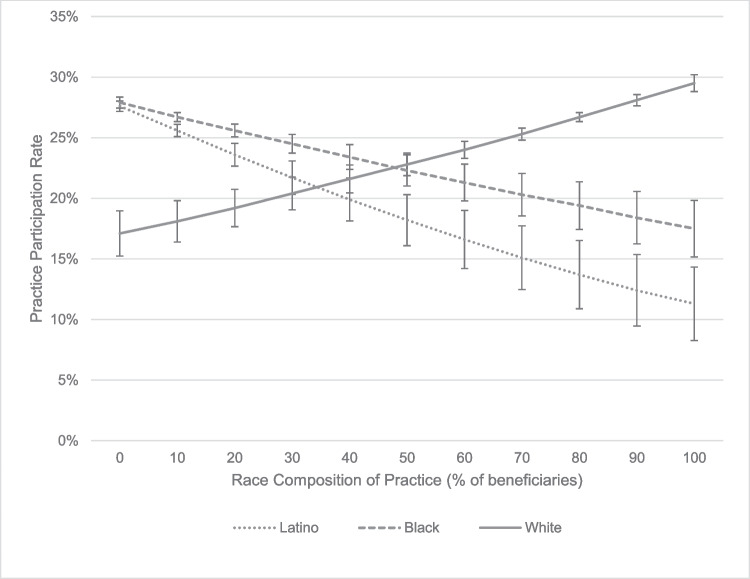
Predicted Comprehensive Primary Care Plus Program participation rate by racial-ethnic composition of primary care practices. Note: Error bars indicate 95% confidence intervals.

Predicted probabilities based on model 3 for practices with varying concentrations of Latino and Black beneficiaries illustrate the lower participation of practices compared to the average participation rate of 26.9% (Fig. 1). When 50% of the beneficiaries are Latino or Black, only 19% and 24%, respectively, are predicted to be CPC+ participants. When the share of Latino or Black beneficiaries is 75%, only 15% and 22%, respectively, are predicted to be CPC+ participants.

Several control variables were also associated with CPC+ participation; practices with higher proportions of dually eligible Medicare and Medicaid beneficiaries (aOR = 0.011 to 0.038), higher proportions of frail elder beneficiaries (aOR = 0.184 to 0.266), and fewer specialists (aOR = 0.443 to 0.428) were less likely to participate in CPC+ compared to practices with lower proportions of these beneficiary populations.

In our sensitivity analysis (model 4), the adjusted odds ratios for practice-level Latino (aOR = 0.32 vs. 0.55) and Black (aOR = 0.62 vs. 0.73) beneficiary concentration slightly attenuated (Table 2, model 4) and were no longer statistically significant. Importantly, 13% of CPC+ practices did not qualify for program participation based on the 125 Medicare FFS beneficiary restriction, highlighting that this criterion was not used consistently to enroll CPC+ practices. The appendix includes detailed information about state practice participation rates for eligible regions (eFigure 5) and the geographic representation of participants (eFigure 6).

## DISCUSSION

We examined whether practices serving relatively high shares of Latino or Black Medicare FFS beneficiaries are less likely to participate in CPC+ in 2021 after controlling for census tract–level poverty, ZIP code level–annual median income, and beneficiaries’ clinical and demographic characteristics. Using multivariable logistic regression analysis, we found that practices serving high shares of Latinos were much less likely to participate in CPC+ compared to practices serving low shares of Latinos, but lower participation among practices with higher shares of Black patients is explained by regional and state participation levels. While the intended goal of CPC+ is to invest in improving primary care practice capabilities, without targeted efforts to engage diverse practices, the program may have the unintended effect of exacerbating disparities given that practices with high proportions of Latino Medicare FFS beneficiaries are less likely to participate in CPC+ and are less likely to have infrastructural support for chronic care management. As a result, racial inequities in quality of care for beneficiaries may increase.

When state fixed effects were included (model 2 to model 3), the odds ratio for the practice share of Black beneficiaries attenuated, while the odds ratio for the practice share of Latino beneficiaries became smaller and more significant. This means that practices with relatively high shares of Black beneficiaries are highly concentrated in states with low CPC+ participation of eligible practices overall. This suggests that Black beneficiary representation could have been improved by increasing practice participation rates in eligible states with high Black Medicare beneficiary concentrations, including Louisiana, Missouri, Ohio, and Tennessee. This is not the case for practices with relatively high proportions of Latino beneficiaries; adding state fixed effects to the model strengthens the association of practice Latino beneficiary concentration and CPC+ participation. The results indicate that practices with high proportions of Latino beneficiaries are much less likely to participate in CPC+ and that improving state or regional participation generally would not have improved the representation of Latino beneficiaries in CPC+. Instead, focused efforts to encourage and recruit practices serving high proportions of Latino beneficiaries across regions would have been needed to improve CPC+’s reach to Latino beneficiaries.

Importantly, the CPC+ program was motivated by a need to help independent primary care practices develop chronic care management processes and improve health information technology capabilities. It is noteworthy then how much independent physician practices continue to be left out of CPC+. Our research study and past studies, however, find that CPC+ has predominantly enrolled practices owned by hospitals, health care systems, and physician group–owned practices.^5,8^ These already advantaged practices have better access to the capital needed to support practice transformation activities beyond the modest CPC+ care management fees compared to independent practices that serve relatively high shares of Latino beneficiaries, which have less access to capital to invest in care management infrastructure. Although CMS may have historically selected practices based on readiness factors to optimize “short term wins” for the program through extensive practice eligibility and participation requirements,^15^ these decisions are associated with low participation of eligible practices with relatively high proportions of Black and Latino beneficiaries. Recent evidence indicates that privately insured patients of CPC+ practices did not experience improved quality or reduced spending^16^ and continuity of care did not improve^17^, suggesting that selecting practices based on extensive readiness factors does not guarantee “short term wins.”

Our research study has some limitations that should be considered when interpreting the results. First, the data used to assess CPC+ participation, characterize practices, and describe beneficiaries were from different years, which may result in misclassification of practices. The study data are the latest proprietary data available at the time that the analyses were conducted. Second, the patient analyses were limited to Medicare FFS beneficiaries as these patients are the focus of CPC+ and central to practice eligibility requirements. The associations we found may differ if Medicare Advantage, Medicaid, or commercial patients were considered in addition to Medicare FFS beneficiaries. Third, unmeasured characteristics of non-CPC+ practices may make them ineligible and could explain differences in CPC+ participation rates. For example, practices participating in TCPI, another CMS-based health care transformation program, are not eligible for CPC+, but TCPI was awarded at a network level instead of the practice level making it difficult to identify TCPI practices. Consequently, our control group may include TCPI practices who were ineligible for CPC+. Fourth, we were not able to examine which selection criteria contribute to lower participation of practices that serve relatively high proportions of Black and Latino beneficiaries. Data on practice electronic health record capabilities and previous experience with practice transformation programs could further explain differences in CPC+ participation between practices based on beneficiary racial-ethnic concentration.^5^ Finally, our sensitivity analysis (model 4) reduced the analytic sample by 30%, decreasing statistical power. The loss of 13% of CPC+ practices for this model highlights the inconsistent application of eligibility criteria by the CPC+ program when selecting practices and the reduced generalizability when limiting analyses to practices with 125 or more beneficiaries.

## CONCLUSION

The CMS CPC+ program’s selection criteria and recruitment efforts left Latino and Black Medicare FFS beneficiaries disproportionately out of the many benefits of primary care practice transformation.^18–22^ We found that the racial-ethnic composition of eligible primary care practices is more strongly associated with CPC+ participation than census tract–level poverty. Our findings and past research^5^ suggest that low Medicare FFS beneficiary volume, low electronic health record capabilities, and limited experiences of engaging in practice transformation may contribute to lower CPC+ participation rates among practices with relatively high concentrations of Black and Latino Medicare FFS beneficiaries. CMS recently committed to advancing equity within future payment and delivery reform models.^9^ As practice eligibility criteria for future innovation models that prioritize equity are considered,^23^ special attention should be given to the racial and ethnic diversity of beneficiaries of enrolled practices to ensure that federally sponsored practice transformation resources can advance racial equity, while improving overall quality and managing total spending. Given the high concentration of Latino and Black beneficiaries in lower-volume practices, CMS should focus on its plan to increase the participation of the practices in practice transformation initiatives in ways that support their missions of providing access to care for minoritized populations.

### Supplementary Information

Below is the link to the electronic supplementary material.

Supplementary file1 (DOCX 278 KB)
