# Supplementary material for: Racial-Ethnic Composition of Primary Care Practices and Comprehensive Primary Care Plus Initiative Participation
Source: J Gen Intern Med. 2023 Mar 20;38(13):2945–52. doi: 10.1007/s11606-023-08160-0 (PMC10593678; doi:10.1007/s11606-023-08160-0)
Supplement: Supplementary file 1 — Supplementary file1 (DOCX 278 KB) [file 11606_2023_8160_MOESM1_ESM.docx]

**Appendix**

**Table of Contents**

- **Supplemental Methods Description**
- **eFigure 1.** Sample Restriction to Eligible Regions of the Comprehensive Primary Care Plus (CPC+) Program
- **eFigure 2.** Identification of Comprehensive Primary Care Plus (CPC+) Practices
- **eFigure 3.** Identification of Comparison (non-CPC+) Practices
- **eFigure 4.** CPC+ Participation Rates in the 18 Eligible Regions
- **eFigure 5.** Geographical Representation of CPC+ Participation Rates in the United States

**Supplemental Methods Description**

Of the 414,349 primary care practices in the IQVIA OneKey, we identified 3,154 practices to be CPC+ participants. We used the *matchit* command in STATA 14.2 to estimate probability of business names in IQVIA matching with a practice name in the CMS CPC+ participant data. Practices with a probability score of 1 are exact matches while practice names with score less than 1 have practice names from each data source that varies in spelling, capitalization, spacing, etc. Manual matching of observations with probability scores less than 1 was conducted by two co-authors and differences were reconciled through discussion. Overall, 97% of CMS participant list matched with IQVIA practice data and non-matched practices were dropped.

We excluded FQHC practices located in the 18 regions (n=2,891) based on Health Resources & Services Administration grantee data because FQHCs are not eligible to participate in CPC+. We restricted the comparison group practices to the adult primary care specialties of family practice, internal medicine, multispecialty group practice, and primary care, excluding specialist practices (n=128,370). We also excluded practices located in counties within CPC+ states that were outside of eligible CPC+ regions (n=5,572), resulting in 12,420 non-CPC+ practices.

We used 2018 Medicare parts A and B claims data to determine diagnoses and spending. U.S. Census data were integrated with claims data to characterize the median annual household income within each beneficiary’s five-digit ZIP Code and whether the beneficiary resided in a Census tract with high poverty (20 percent or more of residents with incomes at or below 100 percent of the federal poverty level). We excluded practices (n=4,006) from the integrated 2019 IQVIA One Key data and 2021 CPC+ practice participant file that did not have any Medicare FFS beneficiaries attributed to them.

To characterize beneficiary sociodemographic and clinical characteristics for each practice, the integrated IQVIA OneKey and CPC+ participation data (3,154 CPC+ practices and 12,420 non-CPC+ practices) were then merged with 2018 beneficiary-level Medicare FFS claims (n=7,264,812). We attributed beneficiaries to practice sites using methods that CMS uses as part of MSSP, which is a well-documented and widely accepted method^1^ and comparable to the method used in the CPC+ program. This method favors assignment of beneficiaries to primary care clinicians over specialists and is based on where patients receive the plurality of their primary care services.

Reference

1. *Medicare Shared Savings Program Shared Losses and Assignment Methodology*. Centers for Medicare and Medicaid Services; 2017. Accessed May 17, 2021. https://www.cms.gov/Medicare/Medicare-Fee-for-Service-Payment/sharedsavingsprogram/Downloads/Shared-Savings-Losses-Assignment-Spec-V5.pdf

**eFigure 1. Sample Restriction to Eligible Regions of the Comprehensive Primary Care Plus (CPC+) Program**

Note: the figure above simplifies the method that we used to restrict our practice sample to the 18 CPC+-eligible states and regions.

**eFigure 2. Identification of Comprehensive Primary Care Plus (CPC+) Practices**

CMS Participant List: Assessed for CPC+ Identification

N = 3,428

Excluded because the practice does not have at least one Medicare FFS beneficiary:

N = 115

Excluded because the practice does not have at least one physician or advanced practice clinician:

N = 6

Excluded because outside the eligible regions:

N = 153

Included: N = 3,269

Included: N = 3,275

I Analytical Sample:

N = 3,154

## Located in the Eligible Regions

## With Attributed Medicare FFS Beneficiaries

## At Least One Physician or Advanced Practice Clinician

**eFigure 3. Identification of Comparison (non-CPC+) Practices**

Number of Practices Unmerged with the CMS Participant List: Assessed for non-CPC+ Identification:

N = 410,921

Excluded because practice was located outside of county FIPS codes that are eligible for CPC+:

N = 5,594

Excluded because practices are not primary care or are FQHCs:

N = 129,954

Excluded because outside the eligible

geographic regions:

N = 262,943

Included: N = 18,024

Included: N = 147,798

I Included: N = 12,430

## Located in the Eligible Regions

## Located Inside CPC+ County FIPS Code

## Primary Care and non-FQHC Practice

## With Attributed Medicare FFS Beneficiaries

Analytical Sample:

N = 8,566

Excluded because practices do not have at least one Medicare FFS beneficiary:

N = 3,864

**eFigure 4. CPC+ Participation Rates in the 18 Eligible Regions**

Note: States with full participation in the CPC+ Initiative are shaded in grey, while states with partial participation in CPC+ based on regional participation are shaded in black. Specifically, (1) New York participation rate only represents the combined overall participation rate in the Greater Buffalo Region and the North Hudson-Capital Region. (2) Kansas and Missouri participation rates only represent the participation rates that are inside the Greater Kansas City Region of Kansas and Missouri. (3) Ohio and Kentucky participation rates only represent the participate rates that are inside the Ohio and Northern Kentucky Region. All the other states represent participation rates for the entirety of its geographical borders.

**eFigure 5. Geographical Representation of CPC+ Participation Rate in the United States**


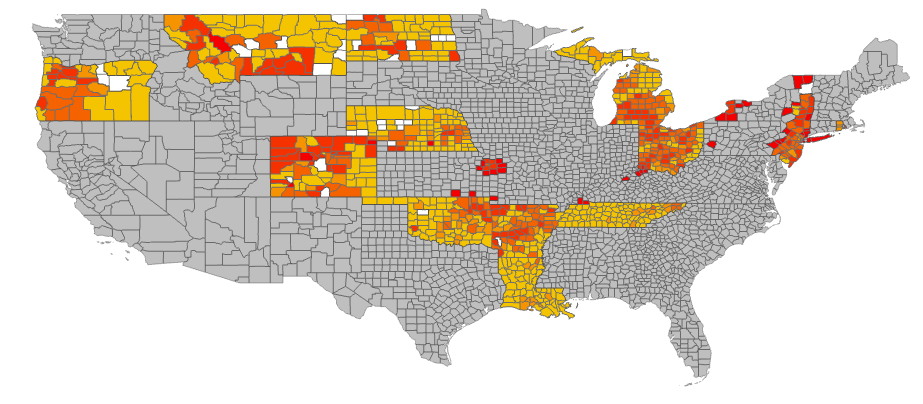


Hawaii


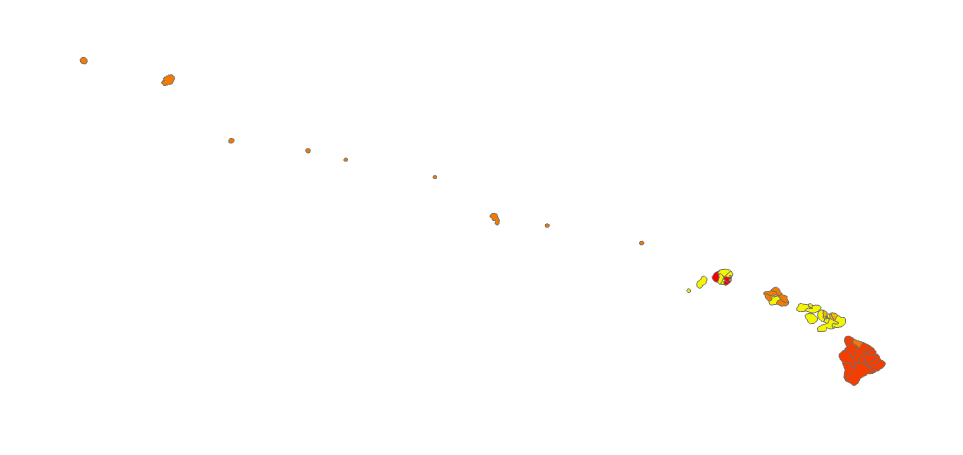

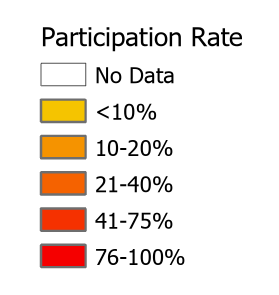


Note: The map above shows the geographical expanse of CPC+ participation by eligible counties. Counties in dark orange have relatively higher CPC+ participation while counties in yellow have relatively lower CPC+ participation. Note that most practices of New York, Ohio, Kentucky, Kansas, and Missouri were not eligible, as only specific regions in these states were eligible. Note that Alaska is an ineligible state and is also omitted from the map. Hawaii is shown in a separate box.
